# Dynamics of Changes in the Gut Microbiota of Healthy Mice Fed with Lactic Acid Bacteria and Bifidobacteria

**DOI:** 10.3390/microorganisms10051020

**Published:** 2022-05-12

**Authors:** Mariya Gryaznova, Yulia Dvoretskaya, Inna Burakova, Mikhail Syromyatnikov, Evgeny Popov, Anastasia Kokina, Evgeny Mikhaylov, Vasily Popov

**Affiliations:** 1 Laboratory of Metagenomics and Food Biotechnology, Voronezh State University of Engineering Technologies, 394036 Voronezh, Russia; mariya-vg@mail.ru (M.G.); dyd16@mail.ru (Y.D.); vitkalovai@inbox.ru (I.B.); e_s_popov@mail.ru (E.P.); nastenka.kokina@mail.ru (A.K.); pvn@vsuet.ru (V.P.); 2 Department of Genetics, Cytology and Bioengineering, Voronezh State University, 394018 Voronezh, Russia; 3 FSBSI All-Russian Veterinary Research Institute of Pathology, Pharmacology and Therapy, 394061 Voronezh, Russia; voronezh81@rambler.ru

**Keywords:** microbiome, probiotics, sequencing, mice, *16S rRNA*, NGS, bifidobacteria, lactic acid bacteria

## Abstract

Probiotics are living microorganisms that provide numerous health benefits for their host. Probiotics have various effects on the body; for example, they change gut microbiota, improve the integrity of the epithelial barrier and have anti-inflammatory effects. The use of probiotic supplements that are based on lactic acid bacteria and bifidobacteria is one of the approaches that are used to balance gut microflora. In our study, we evaluated the effects of supplements, which were based on members of the *Lactobacillaceae* family and bifidobacteria, on the gut microbiome of healthy mice using the *16S rRNA* sequencing method. The data that were obtained demonstrated that when mice received the probiotic supplements, statistically significant changes occurred in the composition of the microbiome at the phylum level, which were characterized by an increase in the number of *Actinobacteriota*, *Bacteroidota*, *Verrucomicrobia* and *Proteobacteria*, all of which have potentially positive effects on health. At the generic level, a decrease in the abundance of members of the *Nocardioides*, *Helicobacter* and *Mucispirillum* genus, which are involved in inflammatory processes, was observed for the group of mice that was fed with lactic acid bacteria. For the group of mice that was fed with bifidobacteria, a decrease was seen in the number of members of the *Tyzzerella* and *Akkermansia* genus. The results of our study contribute to the understanding of changes in the gut microbiota of healthy mice under the influence of probiotics. It was shown that probiotics that are based on members of the *Lactobacillaceae* family have a more positive effect on the gut microbiome than probiotics that are based on bifidobacteria.

## 1. Introduction

The gastrointestinal tract of mammals contains a complex community of microorganisms. The taxonomy of this community can vary significantly from individual to individual, but the main microbiome has a high functional redundancy and plays an important role in maintaining the health of its host [1].

It is well known that representatives of probiotic bacteria, such as lactic acid bacteria and bifidobacteria, are beneficial for human and animal health [2]. Their use in food has various positive effects, including the prevention of infections [3], the modulation of lipid metabolism to prevent and control obesity, reductions in allergic symptoms and the alleviation of diarrhea [4] and constipation, because they stimulate the immune system of the mucous membrane and the systemic immune response and/or change the gut microbiota [5].

The use of probiotic supplements, which contain members of the *Lactobacillaceae* family and the *Bifidobacterium* genus, is one of the modern approaches that are used to positively change the balance of gut microflora [6]. However, the effects of probiotic interventions on the microbiome as a whole cannot be fully assessed without a study on healthy organisms.

The effects of probiotics on the composition, diversity and function of gut microbiota has been studied using various tools and methods, ranging from targeted culture-dependent methods to metagenomic sequencing [7]. Previous studies have demonstrated significant changes in microbiota after probiotic treatment [8]. For example, Khan et al. showed the beneficial effects of *Lactobacillus plantarum* on the microbiome of mice, which were demonstrated by an increase in the beneficial bacteria that are associated with the production of short-chain fatty acids and a reduction in pro-inflammatory cytokines [9]. The addition of *L. fermentum* prevented hyperlipidemia, inflammation and oxidative stress in the colon and heart tissues of rats that were fed a high-fat diet. Studies have also shown that *Lactobacillus acidophilus* can inhibit pathogens and modulate immunity [10]. It has been shown that *B. bifidum* can have beneficial effects on cholesterol metabolism through the modulation of gut microbiota, as well as alleviating the symptoms of irritable bowel syndrome [11]. The beneficial effects of *B. longum* are based on a high carbohydrate metabolism followed by the production of acetate, which is a short-chain fatty acid that activates the barrier function of the intestinal epithelium of the host [12]. *B. adolescentis* is recognized as one of the dominant anaerobes that are found in adults and is considered to be beneficial to health. Some strains can regulate the *Proteobacteria* to *Bacteroidetes* ratio in gut microbiota and inhibit NF-κB activation in the colon [13,14].

Despite the fact that the effects of probiotic bacteria on organisms with various pathologies have been widely studied, there have been very limited studies on the effects of probiotic bacteria on the gut microbiome of healthy organisms. Thus, it has been shown that the addition of a probiotic of one species (*Bifidobacterium infantis*) does not affect the diversity and composition of the microbiome of the gastrointestinal tract of a healthy adult [15]. The intake of a yogurt with *Bifidobacterium animalis* subsp. lactis BB-12 increases the presence of beneficial bacteria, such as those in the *Bifidobacterium* genus, *Slackia isoflavoniconvertens* and *Adlercreutzia equolifaciens* [16]. *L. rhamnosus* CNCM I-4036 significantly increases the abundance of members of the *Lactobacillus* genus in the gut of a healthy adult [17]. No significant changes were found in the microbiological composition of the gut of healthy Japanese people following the consumption of probiotics that contained either *Lactobacillus* or *Bifidobacterium* strains [18]. Similarly, no effects of probiotics (*Lactobacillus rhamnosus* (LGG^®^) and *Bifidobacterium animalis* subsp. *lactis* (BB-12^®^)) were found on the bacterial composition of the gut of Danish infants [19].

The effects of probiotics on the bacterial composition of the gut of healthy animals have also been relatively poorly studied. However, there have been studies that show the effectiveness of the use of probiotic supplements for preventive purposes [20,21,22,23]. For example, Yang et al. showed that the combination of *Cochlearius cochlearium* and *L. acidophilus* has beneficial effects on body weight control and glucose homeostasis in high-fat DIO mice [24]. However, the dynamics of the changes in gut microbiota during long-term feed intake with the addition of probiotic bacteria have not been studied. The aim of this research was to study the effects of two types of probiotics (one based on lactic acid bacteria and the other on bifidobacteria) on the microbiome composition of mouse feces, depending on the duration of their use as a dietary supplement, using the next-generation sequencing method (NGS).

## 2. Materials and Methods

### 2.1. Experiment Design

Our study involved six-week-old *Mus musculus* of the C57BL/6 line. The mice were delivered from the farm of the Scientific Center for Biomedical Technologies (Stolbovaya branch, Russia). Each mouse was kept in a separate cage in a temperature-controlled room at 25 ± 2 °C and with a 12 h light–dark cycle. All animals had free access to water and were fed an ad libitum diet. The animals were fed a standard laboratory diet (Ssniff-Spezialdiäten GmbH, Soest, Germany). The composition of the diet is presented in Supplementary Materials File S1 (diet composition).

Initially, commercial probiotic bacteria (White Lily Ltd., Voronezh, Russia) were used. Pure bacterial cultures were deposited in the scientific microbiological museum of the Department of Biochemistry and Biotechnology at the Voronezh State University of Engineering Technologies and the following numbers were assigned: *Bifidobacterium bifidum*, *VSUET01*; *Bifidobacterium longum*, *VSUET02*; *Bifidobacterium adolescentis*, *VSUET03*; *Lactobacillus acidophilus*, *VSUET12*; *Lactiplantibacillus plantarum*, *VSUET13*; and *Limosilactobacillus fermentum*, *VSUET14*. Bacteria were added into 100 mL of sterilized skimmed milk. To activate the microbial cells, the concentrate was thoroughly mixed and kept for 4.0 h at a temperature of 37 ± 1 °C. Then, 1 and 2 h after the start of activation, the bacterial suspension was stirred again (by shaking) to encourage the distribution of bacterial cells throughout the solution. Immediately after activation, the resulting activated concentrate was introduced into pasteurized (92 ± 2 °C, 2–8 min exposure) and cooled (37–42 °C) skimmed milk by stirring. The concentration of microorganisms in the finished product was brought to 10^8^ CFU/mL. The prepared cultures of bacteria were stored at −4 °C for no more than 3 days. The number of viable cells of lactic acid bacteria was calculated using the MRS agar.

The feed was crushed to a particle size of no more than 5 mm and then mixed with the prepared bacterial cultures in sterile conditions at a ratio of 9 g of feed to 1 g of bacterial culture. The feed in cage was replaced with freshly prepared feed every day.

**Figure 1:**
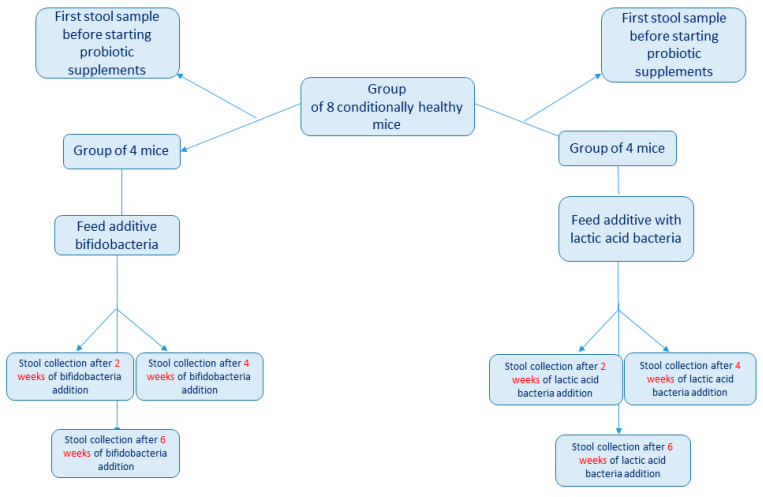
Design of the experiment to collect biomaterial for sequencing. Lactic acid bacteria refers to feed with the addition of lactic acid bacteria species and bifidobacteria refers to feed with the addition of *Bifidobacterium* species.

The test animals were divided into two main research groups, each of which included four healthy male mice. The first stool collection was carried out before the addition of the probiotic supplements into the feed for all mice. Then, the first group received supplements with a mixture of bifidobacteria (*B. bifidum*, *B. longum* and *B. adolescentis*) and the second group received supplements with a mixture of *Lactobacillaceae* species (*Lactobacillus acidophilus*, *Lactiplantibacillus plantarum* and *Limosilactobacillus fermentum*). The concentration of each bacterial species in the supplement was 10^7^ CFU per gram of feed. Then, 0.2 ± 0.01 g of fecal matter was collected from each mouse after two, four and six weeks for the subsequent estimation of changes in the composition of their gut microbiome under the influence of probiotics (Figure 1). All fecal samples were immediately placed into sterile 1.5-mL Eppendorf tubes and stored at −80 °C until DNA isolation.

### 2.2. Sequencing

DNA was extracted from each fecal sample using the ZymoBiomics DNA Miniprep Kit (Zymo Research, Los Angeles, CA, USA), according to the protocol. The V3 region of the *16S rRNA* gene was selected for library preparation and further sequencing using the Ion Torrent PGM platform. The targeted amplification of this region was performed using the universal primers 337F (5′-GACTCCTACGGGAGGCWGCAG-3′) and 518R (5′-GTATTACCGCGGCTGCTGG-3′) and the 5X ScreenMix-HS Master Mix Kit (Evrogen, Moscow, Russia). The amplification protocol consisted of the following temperature regimes: 94 °C for 4 min; 37 cycles of 94 °C for 30 s, 53 °C for 30 s and 72 °C for 30 s; and then, final elongation at 72 °C for 5 min. After that, the PCR products were purified using AMPureXP magnetic particles (Beckman Coulter, Brea, CA, USA) and proceeded directly into the preparation of sequencing libraries.

Libraries were prepared using the NEBNext Fast DNA Library Prep Kit (New England Biolabs, Ipswich, MA, USA), according to the manufacturer’s instructions. The resulting libraries were barcoded using NEXTflex DNA Barcodes (Ion Torrent; 64 adapters; PerkinElmer, Inc., Waltham, MA, USA). After the ligation of the adapters, the purification of the finished libraries using the AMPureXP magnetic particles (Beckman Coulter, Brea, CA, USA) was repeated.

Sequencing was performed on the IonTorrent PGM platform using the Ion PGM Hi-Q View OT2 Kit and the Ion PGM Hi-Q View Sequencing Kit, according to the protocol using the Ion 318™ Chip v2 BC chip (ThermoFisher Scientific, Madison, WI, USA).

### 2.3. Statistical Analysis

Based on the results of sequencing, BAM files were obtained for each sample, which were then converted into the FastQ format using the FileExporter plugin and further analyzed using the R programming language in the RStudio environment (VSEARCH v.2.8.2 software). For all reads, low-quality reads were filtered out using the maximum expected error threshold of 1.0 (DADA2 package). We also trimmed the reads to the optimal equal length and subjected them to demultiplexing. After preparatory manipulations, dereplication was carried out and we achieved the combination of all identical readings into unique sequences and proceeded to search for the operational taxonomic units (OTUs) using the UNOISE2 algorithm. The taxonomy was determined to the genus using the SILVA database, version 132 (https://www.arb-silva.de/, accessed 14 March 2022). For taxonomy assignment, a maximum threshold of 100% identity was set with variant amplicon sequences.

A statistical comparison of the relative abundance in each study group was performed in the RStudio environment using the DeSEQ2 R package, which employed the generalized linear modelling (GLM) method [25]. In our study, we compared the changes in the microbiome of a group of mice before adding lactic acid bacteria to their feed (point 0) and then again after two (point 1), four (point 2) and six weeks (point 3). We carried out the same comparisons for a group of mice that were fed with bifidobacteria supplements. The p values were obtained using the Wald test. The results were expressed as the mean ± standard error of the mean (SEM). The alpha diversity for each group was calculated using the Shannon index.

### 2.4. Ethical Statements

The experiments on the animals were approved by the Ethics Commission of the FSBSI “All-Russian Veterinary Research Institute of Pathology, Pharmacology and Therapy” (FSBSI “ARVRIPP&T”) (protocol number 7-12/21, 15 December 2021).

## 3. Results

**Figure 2:**
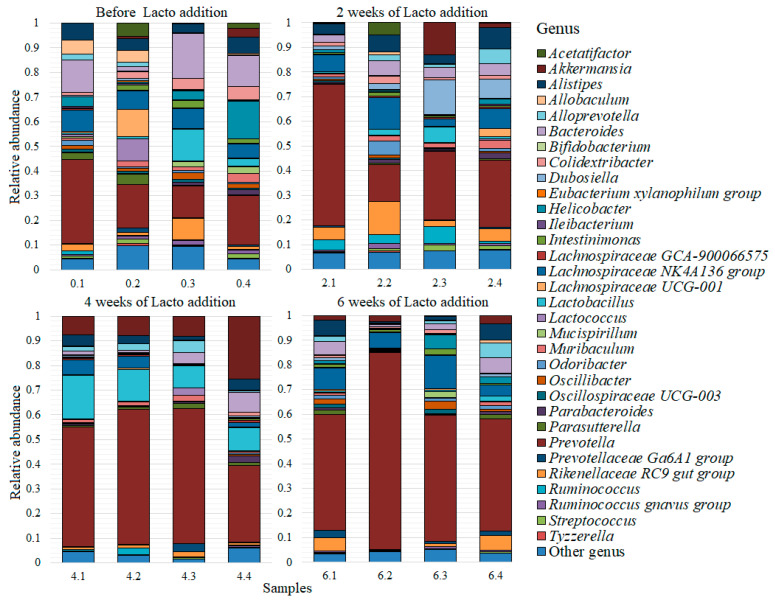
Most common bacterial genera for the group of mice that was supplemented with lactic acid bacteria. Lacto addition refers to feed with the addition of lactic acid bacteria (members of the *Lactobacillaceae* family).

**Figure 3:**
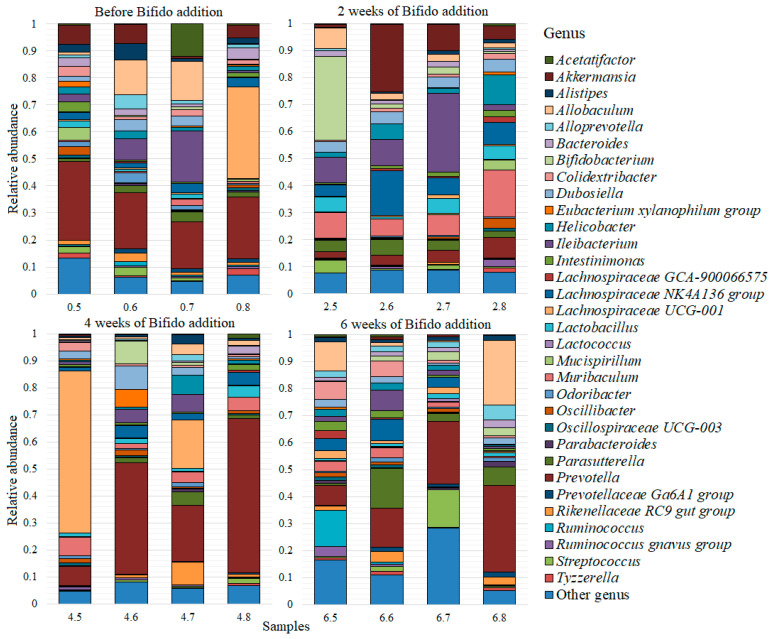
Most common bacterial genera for the group of mice that was supplemented with bifidobacteria. Bifido addition refers to feed with the addition of bifidobacteria.

As a result of the analysis of 32 fecal samples from mice from before and after two, four and six weeks of the addition of two probiotic supplements, a total of 81,385 unique reads were obtained, which corresponded to 99 genera of bacteria. A complete list of the discovered genera is presented in Supplementary Materials (Table S1). All genera that had an average abundance of less than 0.005 were combined into the “Other” group, thus forming a list of the 32 most common genera, which is presented for each sample from the lactic acid bacteria (Figure 2) and bifidobacteria (Figure 3) groups.

For the group of mice that received lactic acid bacteria, the most common genus was *Prevotella*, with an abundance of 0.39 ± 0.04. The next in order of abundance were: *Lachnospiraceae NK4A136 group* (0.07 ± 0.01); *Bacteroides* (0.06 ± 0.01); *Lactobacillus* (0.05 ± 0.01); *Akkermansia* (0.05 ± 0.02); *Alistipes* (0.05 ± 0.01); *Rikenellaceae RC9 gut group* (0.04 ± 0.01); *Helicobacter* (0.02 ± 0.01); *Alloprevotella* (0.02 ± 0.004); *Dubosiella* (0.02 ± 0.01); *Colidextribacter* (0.02 ± 0.004); and *Muribaculum* (0.02 ± 0.002). The average numbers of *Ruminococcus, Parasutterella*, *Lachnospiraceae UCG-001*, *Odoribacter*, *Oscillibacter* and *Intestinimonas* were within 0.01–0.02. The average number of other genera was less than 0.01.

For the group of mice that received bifidobacteria, the most common genus was also *Prevotella* (0.2 ± 0.04) and the second largest was *Lachnospiraceae UCG-001* (0.08 ± 0.04). However, the further distribution of genus was significantly different from that of the group of mice that received lactic acid bacteria. The remaining genera of bacteria were distributed, in terms of abundance, as follows: *Ileibacterium* (0.07 ± 0.02); *Allobaculum* (0.05 ± 0.02); *Lachnospiraceae NK4A136 group* (0.05 ± 0.01); *Muribaculum* (0.05 ± 0.01); *Akkermansia* (0.04 ± 0.02); *Parasutterella* (0.04 ± 0.01); *Bifidobacterium* (0.04 ± 0.02); *Dubosiella* (0.03 ± 0.01); and *Helicobacter* (0.03 ± 0.01). The average relative abundance of the genera *Lactobacillus*, *Streptococcus*, *Colidextribacter*, *Bacteroides*, *Rikenellaceae RC9 gut group*, *Alistipes*, *Alloprevotella*, *Intestinimonas*, *Oscillibacter*, *Ruminococcus*, *Acetatifactor* and *Odoribacter* was within 0.01–0.02 and the average number of other genera was less than 0.01.

**Figure 4:**
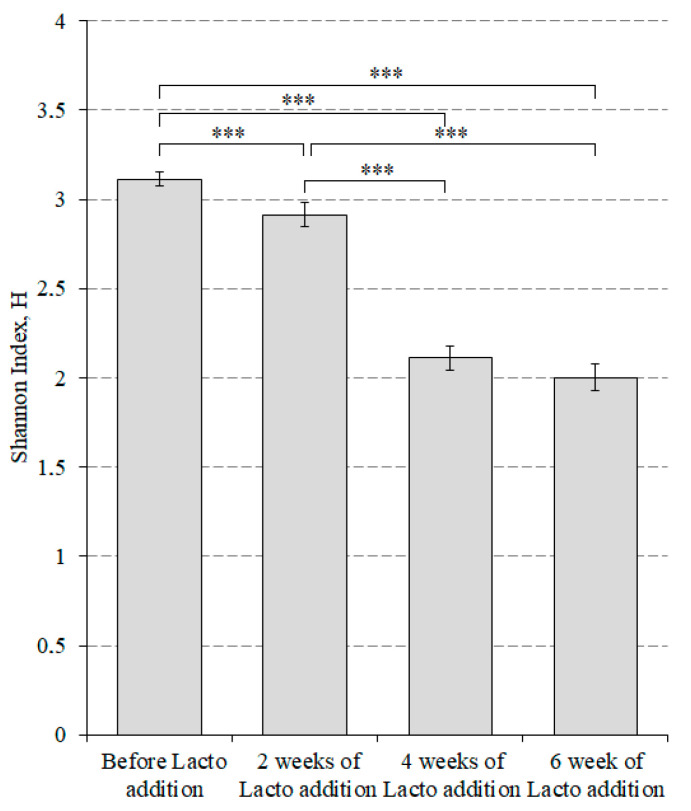
Alpha diversity index for the group of mice that received lactic acid bacteria. *** *p* < 0.001. Lacto addition refers to feed with the addition of lactic acid bacteria.

**Figure 5:**
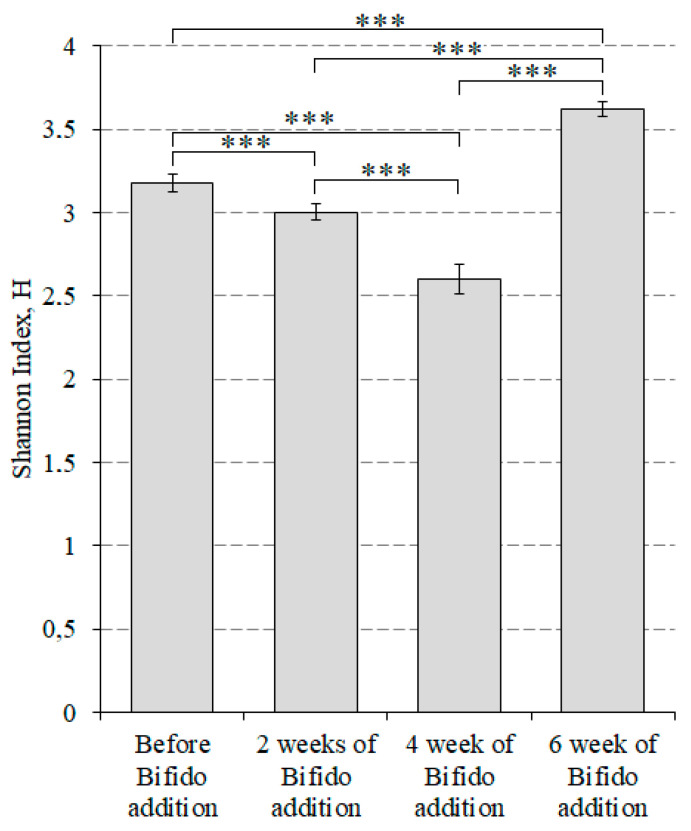
Alpha diversity index for the group of mice that received bifidobacteria. *** *p* < 0.001. Bifido addition refers to feed with the addition of bifidobacteria.

In addition, we estimated the way in which changes in alpha diversity occurred in mice that received lactic acid bacteria (Figure 4) and bifidobacteria (Figure 5) using the Shannon index.

Figure 4 shows that the maximum value of alpha diversity in the group of mice before the addition of members of *Lactobacillaceae* family was 3.11 ± 0.04: two weeks later, it was 2.92 ± 0.06; four weeks later, it was 2.11 ± 0.07; and six weeks later, it was 2.00 ± 0.08. Only the difference between the fourth and sixth weeks was not statistically significant.

Until the fourth week, we observed a decrease in alpha diversity. Before taking bifidobacteria, the indicator was 3.18 ± 0.05. Then, two weeks later, it became 3.0037 ± 0.0474 and by the fourth week, it reached 2.60 ± 0.09. However, after six weeks, the alpha diversity reached its maximum value of 3.62 ± 0.05.

A phylogenetic analysis of the microbiome of the studied groups at the phylum level also revealed several differences in bacterial composition. In the mice that received members of the *Lactobacillaceae* family during the feeding, the phylum *Bacteroidota* increased: 0.48 ± 0.06 before the addition of the probiotic; 0.58 ± 0.07 after two weeks of supplements; 0.62 ± 0.05 after four weeks for supplements; and 0.73 ± 0.05 after six weeks of supplements. At the same time, the phylum *Firmicutes* decreased: 0.41 ± 0.08 before the addition of the probiotic; 0.35 ± 0.05 after two weeks of supplements; 0.23 ± 0.03 after four weeks of supplements; and 0.20 ± 0.04 after six weeks of supplements. These two phyla accounted for the main share of the bacterial composition of the gut of the mice. *Bacteroidota* remained the dominant phylum throughout the lactic acid bacteria feeding.

In the mice that received bifidobacteria during the study, an uneven distribution of bacterial phyla was observed. As in the lactic acid bacteria-treated mice, the two main phyla throughout the study were *Firmicutes* (the abundance of which was 0.51 ± 0.06 before probiotic addition, 0.49 ± 0.05 after two weeks of supplements, 0.47 ± 0.12 after four weeks of supplements and 0.48 ± 0.09 after six weeks of supplements) and *Bacteroidota* (0.37 ± 0.04 before the addition of the probiotic, 0.19 ± 0.03 after two weeks of supplements, 0.44 ± 0.11 after four weeks of supplements and 0.34 ± 0.06 after six weeks of supplements).

We also estimated the statistical significance of differences in the microbiome composition at the phylum level after two, four and six weeks of lactic acid bacteria feeding with a control group of mice. It was found that after two weeks of feeding with probiotics, statistically significant changes were observed for two bacterial phyla: *Campylobacterota*, the abundance of which decreased 2.71-fold, and *Patescibacteria*, the abundance of which increased 4.07-fold compared to the control group (*p* < 0.01 in both cases).

**Figure 6:**
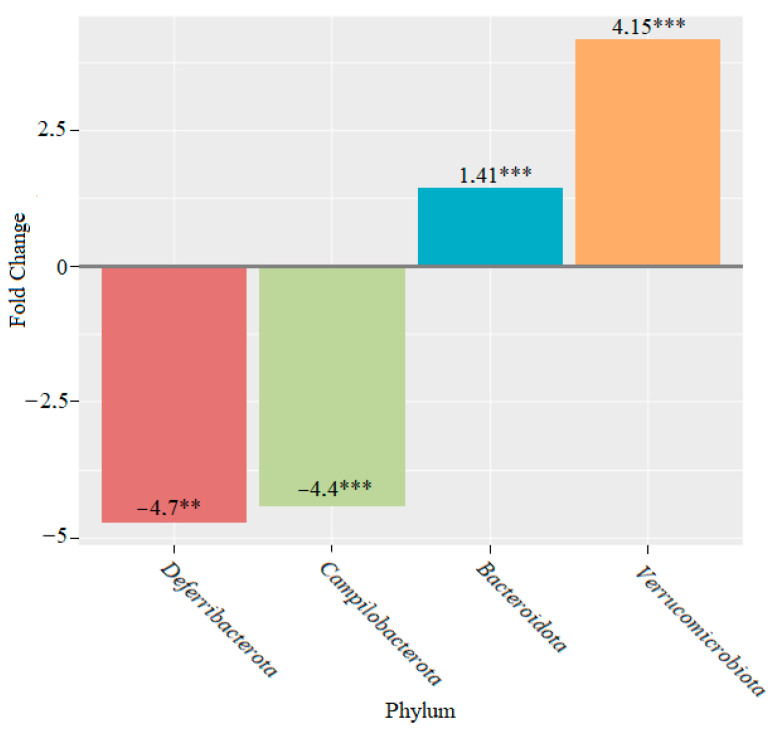
Microbiome changes at the phylum level in mice that were supplemented with lactic acid bacteria for four weeks (** *p* < 0.01; *** *p* < 0.001).

After four weeks of feeding with lactic acid bacteria, statistically significant changes were observed for the following phyla: *Bacteroidota,* which increased 1.41-fold (*p* < 0.001); *Verrucomicrobiota*, which increased 4.15-fold (*p* < 0.001); *Campylobacterota*, which decreased 4.4-fold (*p* < 0.001); and *Deferribacterota*, which decreased 4.7-fold (*p* < 0.01) (Figure 6).

**Figure 7:**
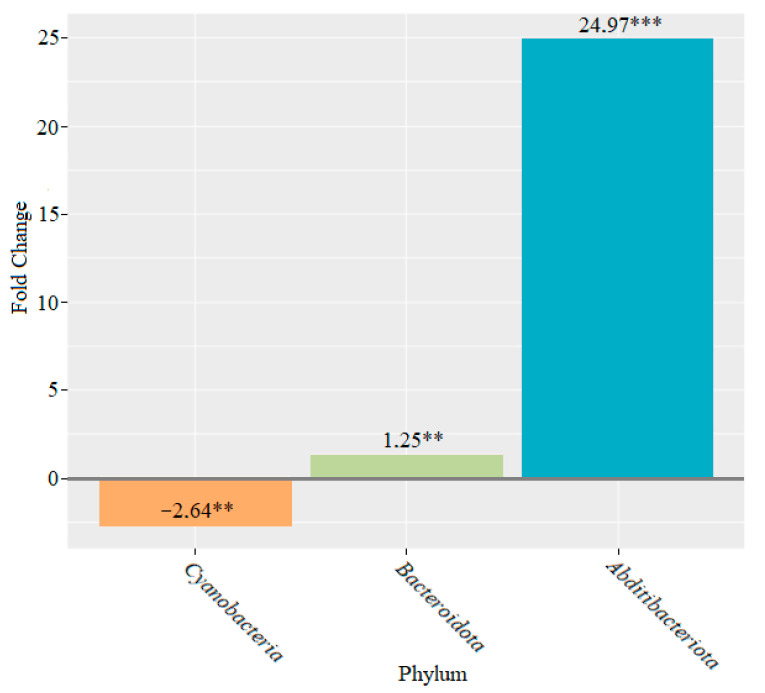
Microbiome changes at the phylum level in mice that were supplemented with lactic acid bacteria for six weeks (** *p* < 0.01; *** *p* < 0.001).

After six weeks of feeding with lactic acid bacteria, statistically significant changes were observed for the following: *Bacteroidota*, which increased 1.25-fold (*p* < 0.01); *Cyanobacteria*, which decreased 2.64-fold (*p* < 0.01); and *Abditibacteriota*, which significantly increased 24.97-fold (*p* < 0.001) (Figure 7).

The statistically significant differences in the microbiome composition at the phylum level that were revealed after two weeks of feeding with a probiotic mixture of bifidobacteria showed an increase in the number of phyla *Actinobacteriota* (4.2-fold (*p* < 0.001)) and *Spirochaetota* (4.26-fold (*p* < 0.01)).

**Figure 8:**
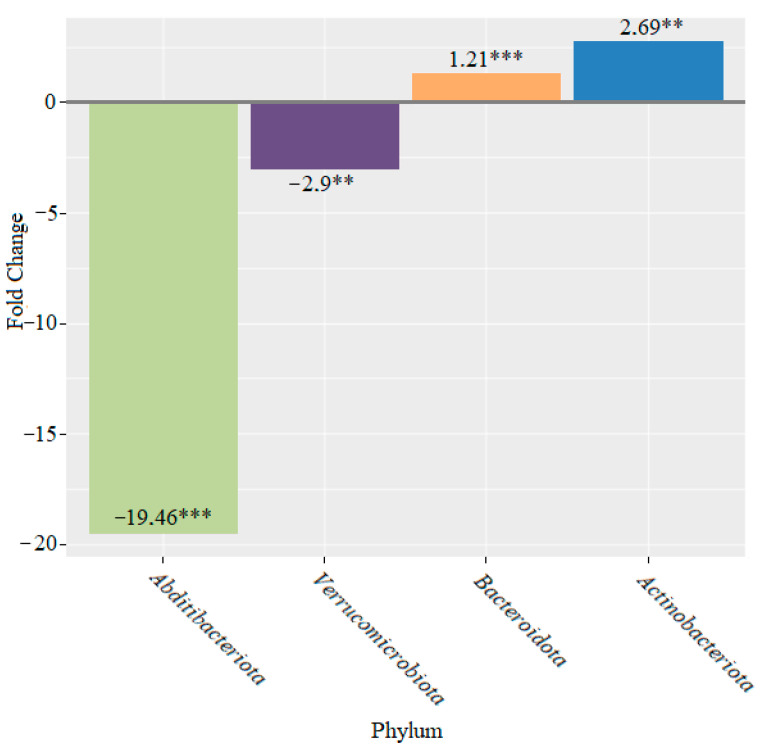
Changes in bacterial composition at the phylum level after four weeks of feeding mice with bifidobacteria (** *p* < 0.01; *** *p* < 0.001).

After four weeks of probiotic feeding, the mice showed a significant decrease in the number of *Abditibacteriota* phyla (19.46-fold (*p* < 0.001)) and *Verrucomicrobiota* (2.9-fold (*p* < 0.01)), as well as an increase in the abundance of the genera *Bacteroidota* (1.21-fold (*p* < 0.001)) and *Actinobacteriota* (2.69-fold (*p* < 0.01)), compared to the control (Figure 8).

**Figure 9:**
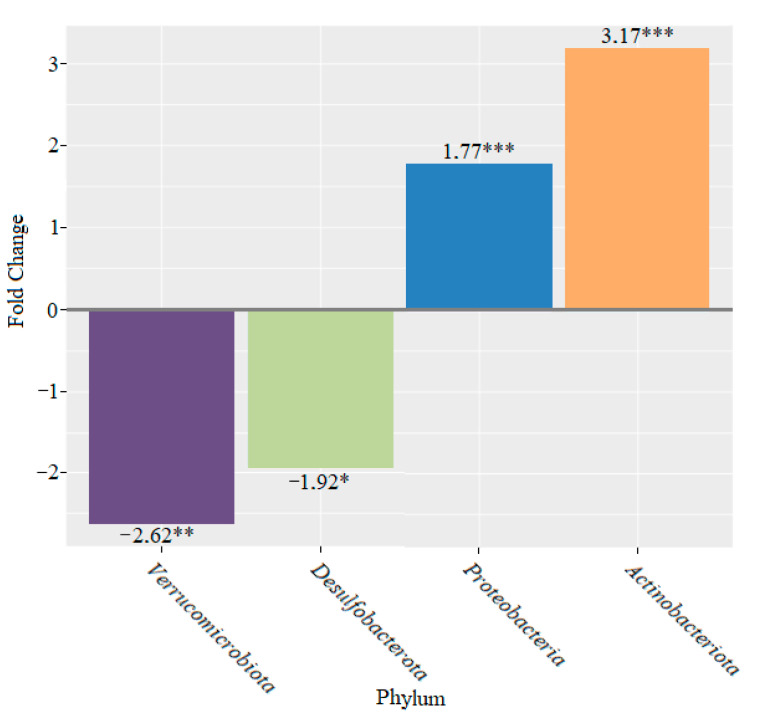
Changes in bacterial composition at the phylum level after six weeks of feeding mice with bifidobacteria (* *p* < 0.05; ** *p* < 0.01; *** *p* < 0.001).

After six weeks of adding bifidobacteria to the feed, we continued to observe a decrease in the phylum *Verrucomicrobiota* (2.62-fold (*p* < 0.01)) and the phylum *Desulfobacterota* (1.92-fold) compared to the control group (*p* < 0.05), while the abundance of the phylum *Proteobacteria* increased 1.77-fold (*p* < 0.001) and *Actinobacteriota* increased 3.17-fold (*p* < 0.001) (Figure 9).

**Figure 10:**
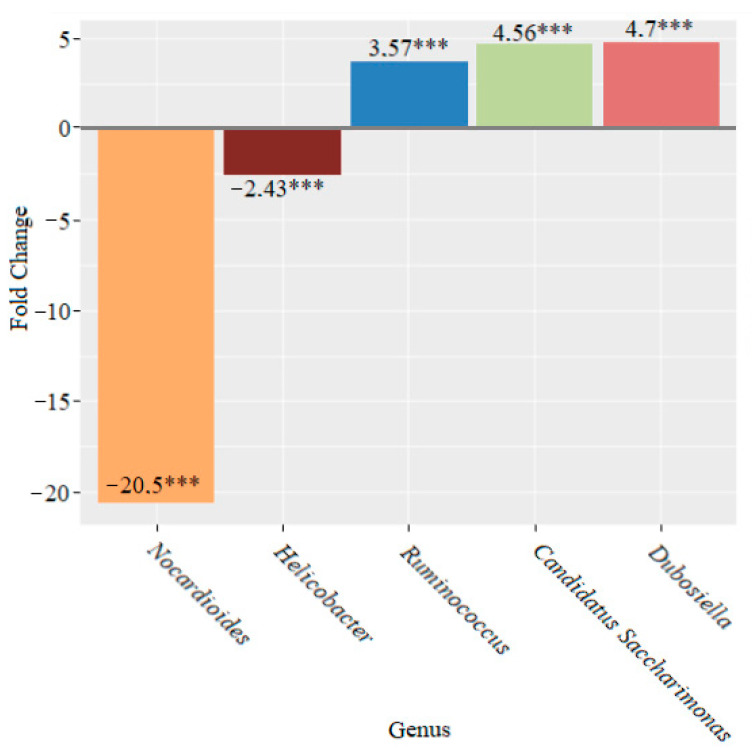
Microbiome changes at the genus level in mice that were supplemented with lactic acid bacteria for two weeks (*** *p* < 0.001).

An analysis of the changes at the genus level in the composition of the microbiome of mice that received lactic acid bacteria showed that after two weeks, there were statistically significant changes, which were characterized by an increase in the abundance of the genera *Ruminococcus* (3.57-fold), *Dubosiella* (4.56-fold) and *Candidatus Saccharimonas* (4.7-fold) and a decrease in *Helicobacter* (2.43-fold) and *Nocardioides* (20.5-fold) compared to the microbiome before the addition of lactic acid bacteria to the feed (*p* < 0.001 in all cases) (Figure 10).

**Figure 11:**
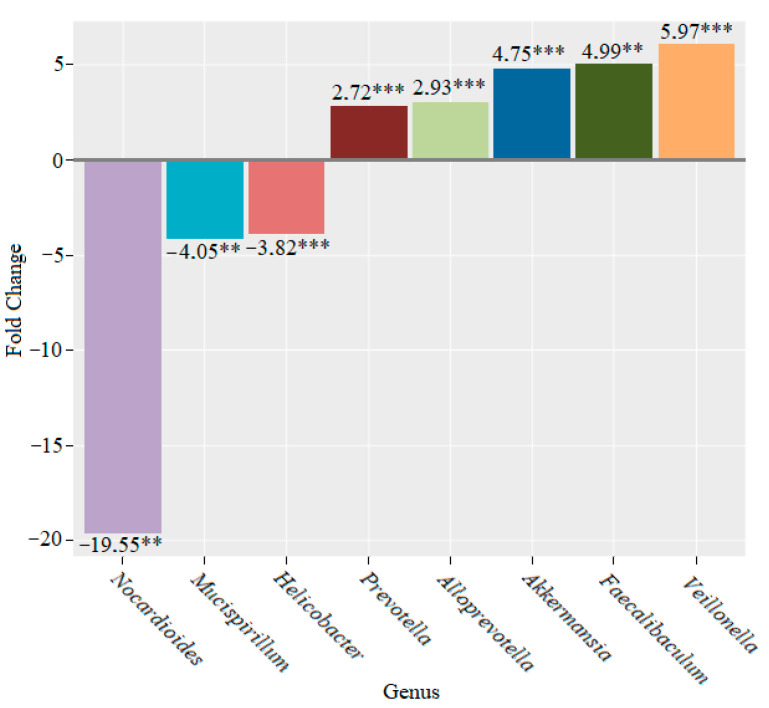
Microbiome changes at the genus level in mice that were supplemented with lactic acid bacteria for four weeks (** *p* < 0.01; *** *p* < 0.001).

Four weeks later, we registered a decrease in the genera *Nocardioides* (19.55-fold (*p* < 0.01)), *Mucispirillum* (4.05-fold (*p* < 0.01)) and *Helicobacter* (3.82-fold (*p* < 0.001)), as well as an increase in the genera *Prevotella* (2.72-fold (*p* < 0.001)), *Alloprevotella* (2.93-fold (*p* < 0.001)), *Akkermansia* (4.75-fold (*p* < 0.001)), *Faecalibaculum* (4.99-fold (*p* < 0.01)) and *Veillonella* (5.97-fold (*p* < 0.001)), compared to the microbiome before the addition of lactic acid bacteria to the feed (Figure 11).

**Figure 12:**
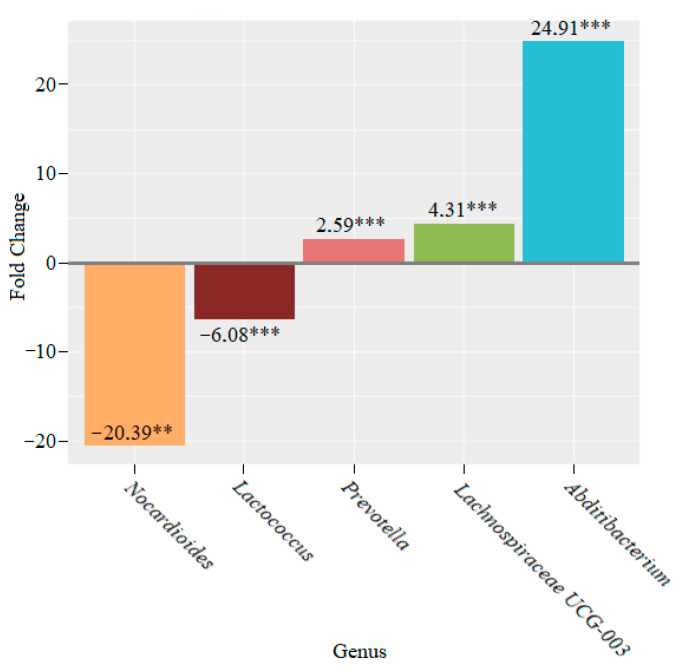
Microbiome changes at the genus level in mice that were supplemented with lactic acid bacteria for six weeks (** *p* < 0.01; *** *p* < 0.001).

After six weeks, in the lactic acid bacteria-fed mice, we observed a 20.39-fold decrease in the *Nocardioides* (** *p* < 0.01) and a 6.08-fold decrease in the *Lactococcus* (*** *p* < 0.001) genera, as well as a 2.59-fold increase in the *Prevotella* (*** *p* < 0.001), a 4.31-fold increase in the *Lachnospiraceae UCG-003* (*** *p* < 0.001) and a 24.91-fold increase in the *Abditibacterium* (*** *p* < 0.001) genera, compared to the control (Figure 12).

**Figure 13:**
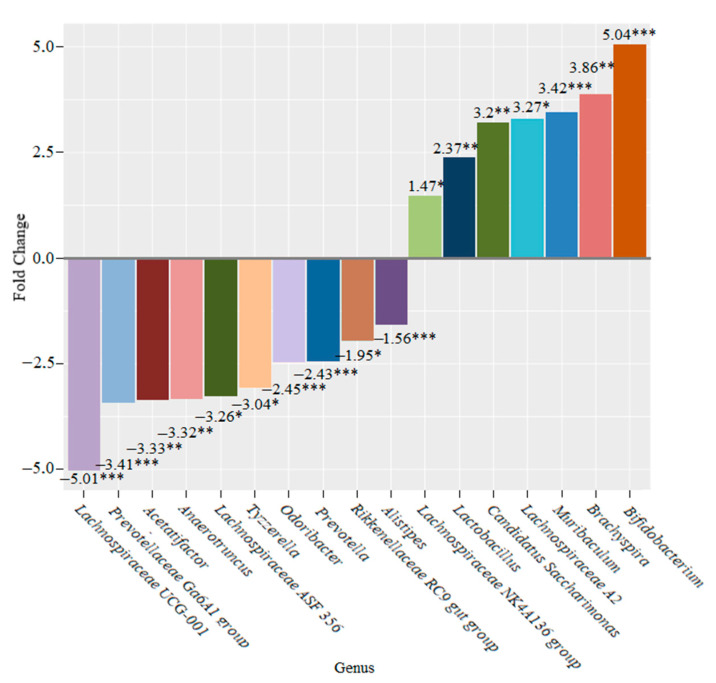
Microbiome changes at the genus level in mice that were supplemented with bifidobacteria for two weeks (* *p* < 0.05; ** *p* < 0.01; *** *p* < 0.001).

Statistically significant decreases were revealed two weeks later in the abundance of *Lachnospiraceae UCG-001* (5.01-fold (*p* < 0.001)), *Prevotellaceae Ga6A1 group* (3.41-fold (*p* < 0.001)), *Acetatifactor* (3.33-fold (*p* < 0.01)), *Anaerotruncus* (3.32-fold (*p* < 0.01)), *Lachnospiraceae ASF356* (3.26-fold (*p* < 0.05)), *Tyzzerella* (3.04-fold (*p* < 0.05)), *Odoribacter* (2.45-fold (*p* < 0.001)), *Prevotella* (2.43-fold (*p* < 0.001)), *Rikenellaceae RC9 gut group* (1.95-fold (*p* < 0.05)) and *Alistipes* (1.56-fold (*p* < 0.001)). Statistically significant increases were observed in the abundance of *Lachnospiraceae NK4A136 group* (1.47-fold (*p* < 0.05)), *Lactobacillus* (2.37-fold (*p* < 0.01)), *Candidatus Saccharimonas* (3.2-fold (*p* < 0.01)), *Lachnospiraceae A2* (3.27-fold (*p* < 0.05)), *Muribaculum* (3.42-fold (*p* < 0.001)), *Brachyspira* (3.86-fold (*p* < 0.01)) and *Bifidobacterium* (5.04-fold (*p* < 0.001)) (Figure 13).

**Figure 14:**
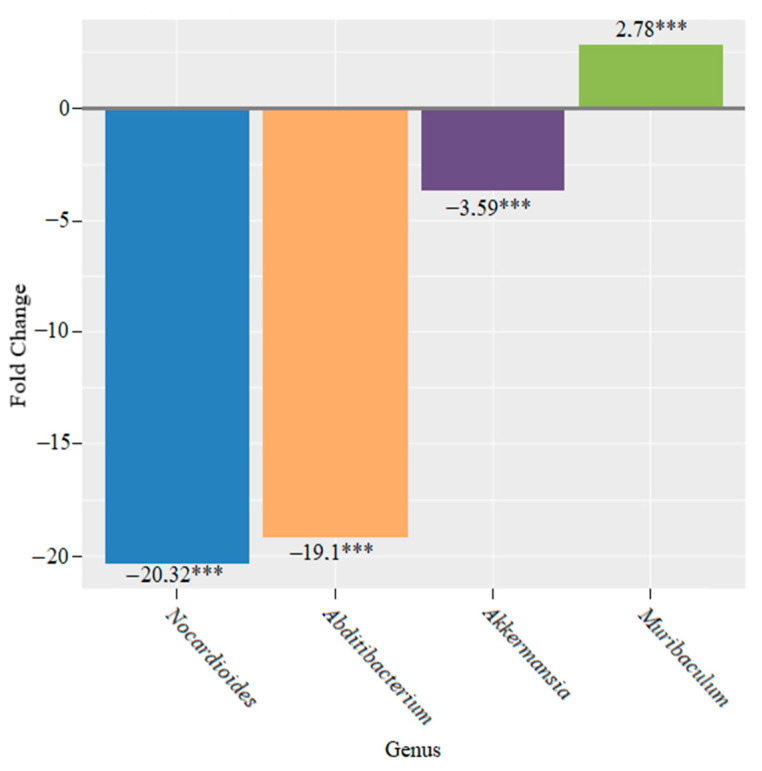
Microbiome changes at the genus level in mice that were supplemented with bifidobacteria for four weeks (*** *p* < 0.001).

Four weeks later, we observed a 20.3-fold decrease in *Nocardioides*, a 19.1-fold decrease in *Abditibacterium*, a 3.59-fold increase in *Akkermansia* and a 2.78-fold increase in *Muribaculum* (*** *p* < 0.001 for all changes) (Figure 14).

After six weeks of probiotic supplementation, the abundance of the genus *Escherichia-Shigella* significantly increased 4.74-fold (*p* < 0.001) compared to the microbiome before the addition of bifidobacteria to the feed.

## 4. Discussion

In this study, we studied the effects of lactic acid bacteria- and bifidobacteria-supplemented diets on the gut microbiome of healthy mice.

Statistically significant changes were observed in two phyla of bacteria after two weeks of feeding with members of the *Lactobacillaceae* family: *Campylobacterota*, the abundance of which decreased, and *Patescibacteria*, the abundance of which increased. We observed an increase in the abundance of the phyla *Bacteroidota* and *Verrucomicrobiota* and a decrease in the abundance of *Campylobacterota* and *Deferribacterota* after four weeks of lactic acid bacteria feeding (Figure 6). After six weeks, the abundance of the genera *Bacteroidota* and *Abditibacteriota* increased, whereas the abundance of the phylum *Cyanobacteria* decreased (Figure 7). 

An increase in the *Campylobacterota* phylum has been associated with the development of gastrointestinal diseases, such as ulcerative colitis (UC) [26]. Additionally, members of the phylum *Helicobacter* are involved in the development of IBD.

The members of the phylum *Bacteroidota* are the main colonizers of the gastrointestinal tract. They play an important role in maintaining the integrity of the interbacterial bonds in the gut. The members of the phylum *Bacteroidota* produce butyrate, which has anti-cancer properties and plays a role in maintaining gut health. In addition, the members of the phylum *Bacteroidota* are involved in the metabolism of bile acids and the transformation of toxic compounds, which also emphasizes their positive role [27]. We observed an increase in their abundance after two weeks of the addition of both types of probiotics.

The phylum *Verrucomicrobia* is a mucin-degrading bacterial community. This phylum is thought to promote gut health and is involved in glucose homeostasis. It can represent 3–5% of the bacterial community and primarily resides in the intestinal mucosa. The abundance of this bacterial phylum significantly reduces body weight and reduces the risk of obesity [28]. The phylum *Verrucomicrobiota* has the potential to induce regulatory immunity and is a possible target for interventions that are aimed at improving regulatory immunity in the gut microbiome [29]. However, in our study, we observed contrasting effects of the probiotics on the abundance of this phylum: the addition of members of the *Lactobacillaceae* family contributed to its increase, while the addition of bifidobacteria led to a decrease.

The phylum *Deferribacterota* is involved in the activation of systemic inflammation in the host organism [30].

According to the available literature data, the members of the phylum *Cyanobacteria* have potential pro-inflammatory, as well as neurotoxic, activity [31].

The influence of members of the phylum *Abditibacteriota* on the host organism has been so far poorly studied. Despite a significant increase in this phylum in the group of mice that was treated with a mixture of lactic acid bacteria for six weeks, this phylum was not included in the list of the most common bacteria, which indicated that there were only trace amounts of microorganisms from this phylum in the studied samples.

The phylum *Desulfobacterota* consists of many organisms that can reduce sulfur compounds through the sulfite reduction pathway for the dissimilation of DsrAB (dissimilatory sulfite reductase). These organisms are involved in the degradation of butyrate through the beta-oxidation pathway, which indicates the involvement of *Desulfobacterota* in the equilibrium of the catabolic reaction [32]. Therefore, it can be assumed that bacteria of this genus can cause inflammatory damage and exacerbate energy metabolism disorders, both of which are pathological signs of diabetes [33].

The phylum *Proteobacteria* is one of the first colonizers of the mammalian gastrointestinal tract. *Proteobacteria* reduce redox potential and also play a key role in the preparation of the gut for subsequent colonization by the strict anaerobes that are necessary for healthy intestinal function [34].

We observed an increase in the genera *Ruminococcus, Dubosiella* and *Candidatus Saccharimonas* and a decrease in *Helicobacter* and *Nocardioides* after two weeks of lactic acid bacteria feeding (Figure 10). After four weeks, we continued to notice declines in the genera *Nocardioides* and *Helicobacter*, whose role in the development of IBD is well known. The population of the genus *Mucispirillum* also decreased. At the same time, the abundance of the members of the genera *Prevotella, Alloprevotella, Akkermansia, Faecalibaculum* and *Veillonella* increased (Figure 11). The data that were obtained demonstrated a significant decrease in the genus *Nocardioides* throughout the feeding of mice with lactic acid bacteria. We also found a decline in the genus *Lactococcus* and an increase in the genera *Prevotella, Lachnospiraceae UCG-003* and *Abditibacterium* (Figure 12).

The members of the genus *Ruminococcus*, which increased after two weeks of feeding with lactic acid bacteria, have ambiguous effects on the host organism. On one hand, there is evidence that the members of this genus are associated with the risk of obesity and their abundance also directly correlates with the development of irritable bowel syndrome (IBS) and the exacerbation of IBD [35]. However, on the other hand, there is also evidence that demonstrates that they can synthesize butyric acid, reduce the risk of cancer, increase immunological protection and have anti-inflammatory effects [36].

According to studies, the genus *Dubosiella* is associated with negative effects on the host organism, which include a direct relationship with the development of obesity [37]. We observed its increase after two weeks of supplemented feeding.

The members of the genus *Candidatus Saccharimonas* produce lactate and acetate. They are associated with inflammatory mucosal disease and can modulate the immune response by downregulating TNF-α gene expression in macrophages. Lower numbers of *Candidatus Saccharimonas* have been noted in acute necrotizing pancreatitis, which is associated with hypertriglyceridemia in rats and mice that have been fed a high-fat diet. This may indicate the potential anti-inflammatory role of *Candidatus Saccharimonas*, an increase in which we observed after two weeks of supplementation with both types of probiotics that were studied [38].

It is known that the members of the genus *Helicobacter* can have multiple negative effects on their host organism, including rodents. Most *Helicobacter* species are long, narrow and slightly curved rods with membrane-covered bipolar flagella. Naturally acquired *Helicobacter* infections have been reported in all commonly used laboratory rodent species. Most *Helicobacter* species in rodents are urease negative and, therefore, preferentially colonize the gut; although in some cases, they may translocate to the liver and biliary system, the stomach or other tissues. The presence of *Helicobacter* spp. deeply affects the host organism. Overall, *Helicobacter* organisms in mice have been associated with IBD and cancers of the breast, liver, stomach and colon [39]. We registered a decrease in this genus after two and four weeks of feeding with lactic acid bacteria.

We also observed a decrease in the abundance of the genus *Nocardioides* at all three test points in the group of mice that was treated with lactic acid bacteria, as well as in the group that was treated with bifidobacteria for four weeks. *Nocardioides* is a rare and poorly studied taxon. There is evidence of this type of increase in the gut of asthmatic mice that were infected with a respiratory syncytial virus [40]. In addition, this genus seems to be inversely correlated with the abundance of the genus *Lactobacillus* and is involved in the development of gut dysbiosis [41].

At the moment, there are no data that describe the role of the genus *Alloprevotella* in the gut of mammals. Our data showed that the increase in the abundance of this genus could be associated with the addition of probiotic bacteria to the diet.

The genus *Akkermansia* has been reported to be correlated with metabolic diseases; however, there are also studies that have proved its beneficial effects on the metabolism of its host [42,43]. We obtained contrasting data for this genus, depending on the type of probiotic that was supplemented: The *Akkermansia* genus increased with the addition of lactic acid bacteria and decreased with the addition of bifidobacteria.

The members of the genus *Faecalibaculum* are known to have antitumor potential in the gut of mice and are capable of producing the metabolites of SCFAs (mainly butyrate) [9,44]. We observed an increase in this genus in mice after four weeks of supplementation with lactic acid bacteria.

The members of the genus *Veillonella* are bacteria that decompose lactate through the formation of propionate SCFAs [45]. *Veillonella* may be sensitive to bile acids since its enrichment is an adaptive response to luminal bile acid deficiency, as in cirrhosis and cholestasis. This adaptation of the microbiota appears to be beneficial as the fermentation of lactate into propionate benefits the microbe by producing additional energy and also benefits the host by eliminating toxic lactate, the accumulation of which is associated with mortality [46].

The bacteria of the genus *Lactococcus* have not been widely recognized for their beneficial effects on gut health and are commonly used in dairy products. However, some data have shown that the members of this genus still have anti-inflammatory efficacy and can reduce oxidative stress in the colonic epithelium of mice that have colitis [47].

It is known that bacteria belonging to the *Lachnospiraceae UCG-003* family can protect against the development of colon cancer by producing butyric acid. At the same time, it has been found that this genus can contribute to the development of diabetes in genetically predisposed mice [48].

With the addition of bifidobacteria, we registered an increase in the abundance of the phyla *Actinobacteriota* and *Spirochaetota* after two weeks. After four weeks, we observed a decrease in the abundance of the phyla *Abditibacteriota* and *Verrucomicrobiota*, as well as an increase in the abundance of the genera *Bacteroidota* and *Actinobacteriota* (Figure 8). After six weeks, we continued to observe a decrease in the phylum *Verrucomicrobiota*. The abundance of the phylum *Desulfobacterota* also decreased, while the abundance of *Proteobacteria* and *Actinobacteriota* increased (Figure 9). We also observed an increase in bifidobacteria belong to the phylum *Actinobacteriota*. Some members of these bacteria are known to have positive effects on the health of the host. It is important to note that a decrease in the levels of bifidobacteria is associated with IBD [49]. Indeed, the use of a combination of bifidobacteria as a supplement causes an accumulation of this phylum of bacteria, which emphasizes the effectiveness of this probiotic.

It is known that some bacteria of the phylum *Spirochaetota* are pathogenic. However, studies have shown a strong decrease in the number of these bacteria in the cases of intestinal diseases [50]. Feeding with bifidobacteria stimulated an increase in the abundance of members of this genus in our experiment.

In the group of mice that were fed bifidobacteria for two weeks, we found significant changes in the composition of their microbiome. These changes were characterized by a decrease in the genus *Lachnospiraceae UCG-001*, *Prevotellaceae Ga6A1 group*, *Acetatifactor*, *Anaerotruncus*, *Lachnospiraceae ASF356*, *Tyzzerella*, *Odoribacter, Prevotella*, *Rikenellaceae RC9 gut group* and *Alistipes* and an increase in *Lachnospiraceae NK4A136 group*, *Lactobacillus*, *Candidatus Saccharimonas*, *Lachnospiraceae A2*, *Muribaculum* and *Bifidobacterium* (Figure 13). After four weeks, we observed a decrease in the abundance of the genera *Nocardioides*, *Abditibacterium* and *Akkermansia* and an increase in the abundance of members of the genus *Muribaculum* (Figure 14). After six weeks, only the abundance of the genus *Escherichia*-*Shigella* increased significantly.

The *Lachnospiraceae UCG-001* bacteria belong to the family *Lachnospiraceae*, the members of which are known to be potent producers of short-chain fatty acids (SCFAs). In addition, one study showed that *Lachnospiraceae UCG-001* may play a significant positive role in the alleviation of type 2 diabetes [51]. However, against the background of a two-week intake of bifidobacteria, the abundance of this genus decreased significantly.

The *Prevotellaceae-Ga6A1* bacteria, which belong to the *Prevotellaceae* family, are bacteria that produce SCFAs and have beneficial effects on the host. However, their abundance also decreased after two weeks of supplementation with bifidobacteria [52].

The bacteria of the genus *Acetatifactor* are strictly anaerobic Gram-positive rods, which produce acetate and butyrate at a ratio of approximately 3:1 [53]. We observed a decrease in this genus after two weeks of probiotic feeding.

*Anaerotruncus* is a recently discovered genus of bacteria, the only species of which is *A. colihominis* [54]. In the literature, no association has been found between this genus and indicators of gut health. In our study, a decrease in this genus was shown for the first time during the intake of bifidobacteria.

The literature that describes *Tyzzerella* is very limited, but there is evidence that this genus increases in patients who have a high-risk profile for cardiovascular disease (CVD). *Tyzzerella* is also overrepresented in patients who have unhealthy diets and Crohn’s disease. Interestingly, numerous recent studies have identified IBD as a risk factor for CVD [55]. Our data demonstrated a decrease in this genus in mice after two weeks of bifidobacteria ingestion.

The bacteria of the genus *Odoribacter* are atypical opportunistic pathogens. We found that the abundance of this genus decreased after two weeks of taking probiotics. The members of *Odoribacter* spp. are associated with several pathologies, such as abdominal abscesses in humans and periodontal problems in other animals. More often, however, their presence appears to be beneficial in preventing some CVDs, such as hypertension [56]. Maintaining the proper number of *Odoribacter* spp. is particularly critical for a healthy gut [57]. A loss of *Odoribacter* spp. leads to a decrease in the availability of SCFAs, which in turn leads to the development of inflammation in the host.

A decrease in the genus *Prevotella* is associated with atrophic gastritis and gastric cancer and an increase in this genus is noted in esophagitis, Barrett’s esophagus, colorectal cancer and in delayed neurodegeneration in mice that have Parkinson’s disease [15,58]. However, in our study, we observed a decrease in the *Prevotella* genus after two weeks of taking bifidobacteria, as well as an increase in this genus after four and six weeks of taking lactic acid bacteria.

The members of the *Rikenellaceae RC9* gut group can neutralize cytotoxic reactive oxygen species and protect cells from oxidative stress, thereby reducing the likelihood of inflammation. In addition, it has been noted that a high-fat diet affects the composition of the microbiome in mouse models, which includes an increase in the abundance of the members of the *Rikenellaceae RC9 gut group* [59]. We observed a decrease in this group of bacteria in mice after only two weeks of dietary supplementation with bifidobacteria.

Some studies have reported the protective role of *Alistipes* against diseases such as colitis, autism spectrum disorders and various fibrotic diseases of the liver and cardiovascular system. Despite this, these bacteria can also play a pathogenic role in diseases such as myalgic encephalomyelitis (also known as chronic fatigue syndrome) and depression [60]. In our study, the abundance of this genus significantly decreased after two weeks of adding bifidobacteria to feed.

Some strains of the genus *Lactobacillus* are probiotic, present in relatively high amounts in the gastrointestinal tract of mice and thought to play a beneficial role in healthy animals [61]. However, despite this, the abundance of this genus declined after two weeks of adding bifidobacteria to feed.

The members of the genus *Brachyspira* are thought to be pathogenic to pigs, birds, dogs and humans and may be a cause of gastroenteritis, UC, acute diarrhea and IBS [62]. It is also known that some of these species can colonize the gut of mice; however, it is not known whether this genus has a pathogenic effect in this case. We observed an increase in the genus *Brachyspira* in mice that were treated with bifidobacteria for two weeks.

The presence of the members of the genus *Bifidobacterium* in the mammalian gut promotes the development of the immune system, helps to maintain the integrity of the intestinal barrier, limits the occurrence of certain gut diseases and provides protection against the multiplication of pathogens [63]. After two weeks of adding this genus to the feed of mice, we registered its direct increase.

*Abditibacterium* is a genus of Gram-negative, aerobic, non-motile and non-spore-forming bacteria, which are extremely resistant to antibiotics and toxic compounds [64]. There have been no data in the literature on the role of this genus in the gut of mammals. However, in our study, data were obtained that showed a significant decrease in the abundance of this genus after four weeks of adding bifidobacteria to feed. At the same time, we observed an increase in the abundance of this genus in mice that received a probiotic mixture of lactic acid bacteria for six weeks.

The genus *Escherichia–Shigella* is extremely diverse and includes a large number of species, ranging from beneficial gut inhabitants to obligate extraintestinal pathogens [65].

## 5. Conclusions

The data that we obtained demonstrated that when mice received probiotic bacteria, there were statistically significant changes in the composition of their gut microbiome at the phylum level. These changes were characterized by an increase in the number of representatives of microorganisms that have potentially positive impacts on health, such as *Actinobacteriota*, *Bacteroidota*, *Verrucomicrobia* and *Proteobacteria*. At the same time, we observed a decrease in the bacterial phylum of *Campylobacteria*, *Deferribacteria*, *Cyanobacteria* and *Desulfobacteria*, which are potentially capable of having negative effects on the health of their host.

A study into the changes in generic microbiome composition during feeding with probiotics showed that when lactic acid bacteria were added to feed, the abundance of the genus *Nocardioides*, the role of which in the gut is not completely clear, decreased. The abundance of the genus *Helicobacter*, the inflammatory activity of which is beyond doubt, decreased. After four weeks of taking a probiotic supplement, a decrease was also observed in the genus *Mucispirillum*, which is involved in inflammatory bowel processes. After six weeks, the genus *Lactococcus* also decreased, which is a potential probiotic but requires further research to prove its anti-inflammatory effectiveness.

Our analysis of the generic composition of the gut microbiota of mice that were fed bifidobacteria for six weeks showed rather ambiguous results. On one hand, we observed that the addition of probiotic bacteria to the diet could actually indirectly modulate the microbiota by increasing the abundance of useful bacteria, such as *Lachnospiraceae* (some groups), *Lactobacillus* and *Bifidobacterium*. However, we also observed a decrease in some bacteria for which their beneficial effects on the host organism have been proven, such as *Prevotellaceae-Ga6A1*, the *Prevotella gut group* and *Rikenellaceae RC9*. We also observed a decrease in the bacteria genera that are associated with an involvement in the development of inflammatory diseases: *Tyzzerella* and *Akkermansia.* For the majority of the genera that we found, it was impossible to unambiguously determine their contribution to the state of gut health, due to either contradictory or limited literature data.

In general, this study contributes to our understanding of the mechanisms of modulation in gut microbiota for various types of probiotic supplements. It was shown that probiotics that are based on *Lactobacillaceae* family members have more pronounced positive effects on the gut microbiome than probiotics that are based on bifidobacteria. Our study provides a rationale for the development of trials that aim to study the effects of probiotics on the composition of the gut microbiome in healthy individuals, which is a topic that has not yet been widely studied.

## Data Availability

Sequencing data are available in the NCBI BioProject database (BioProjectID: PRJNA822353).

## Supplementary Materials

The following supporting information can be downloaded at https://www.mdpi.com/article/10.3390/microorganisms10051020/s1, Table S1: The abundance of microorganisms in the samples. File S1: Diet composition.

## References

[1] Grazul H., Kanda L.L., Gondek D. (2016). Impact of probiotic supplements on microbiome diversity following antibiotic treatment of mice. Gut Microbes.

[2] Yamanaka T., Helgeland L., Farstad I.N., Fukushima H., Midtvedt T., Brandtzaeg P. (2003). Microbial colonization drives lymphocyte accumulation and differentiation in the follicle-associated epithelium of Peyer’s patches. J. Immunol..

[3] Weizman Z., Asli G., Alsheikh A. (2005). Effect of a probiotic infant formula on infections in child care centers: Comparison of two probiotic agents. Pediatrics.

[4] De Vrese M., Marteau P.R. (2007). Probiotics and prebiotics: Effects on diarrhea. J. Nutr..

[5] Makioka Y., Tsukahara T., Ijichi T., Inoue R. (2018). Oral supplementation of *Bifidobacterium longum strain BR-108* alters cecal microbiota by stimulating gut immune system in mice irrespectively of viability. Biosci. Biotechnol. Biochem..

[6] Collins M.D., Gibson G.R. (1999). Probiotics, prebiotics, and synbiotics: Approaches for modulating the microbial ecology of the gut. Am. J. Clin. Nutr..

[7] Kim J.K., Choi M.S., Jeong J.J., Lim S.M., Kim I.S., Yoo H.H., Kim D.H. (2018). Effect of Probiotics on pharmacokinetics of orally administered acetaminophen in mice. Drug Metab. Dispos..

[8] Khan M.T., Nieuwdorp M., Bäckhed F. (2014). Microbial modulation of insulin sensitivity. Cell Metab..

[9] Khan I., Wei J., Li A., Liu Z., Yang P., Jing Y., Chen X., Zhao T., Bai Y., Zha L. (2022). *Lactobacillus plantarum* strains attenuated DSS-induced colitis in mice by modulating the gut microbiota and immune response. Int. Microbiol..

[10] Li Z., Wang W., Liu D., Guo Y. (2018). Effects of *Lactobacillus acidophilus* on the growth performance and intestinal health of broilers challenged with *Clostridium perfringens*. J. Anim. Sci. Biotechnol..

[11] Wang L.J., Yang C.Y., Kuo H.C., Chou W.J., Tsai C.S., Lee S.Y. (2022). Effect of *Bifidobacterium bifidum* on Clinical Characteristics and Gut Microbiota in Attention-Deficit/Hyperactivity Disorder. J. Pers. Med..

[12] Sugahara H., Odamaki T., Fukuda S., Kato T., Xiao J.Z., Abe F., Kikuchi J., Ohno H. (2015). Probiotic *Bifidobacterium longum* alters gut luminal metabolism through modification of the gut microbial community. Sci. Rep..

[13] Guo Y., Xie J.P., Deng K., Li X., Yuan Y., Xuan Q., Xie J., He X.M., Wang Q., Li J.J. (2019). Prophylactic effects of *Bifidobacterium adolescentis* on anxiety and depression-like phenotypes after chronic stress: A role of the gut microbiota-inflammation axis. Front. Behav. Neurosci..

[14] Wang L., Hu L., Xu Q., Yin B., Fang D., Wang G., Zhao J., Zhang H., Chen W. (2017). *Bifidobacterium adolescentis* Exerts Strain-Specific Effects on Constipation Induced by Loperamide in BALB/c Mice. Int. J. Mol. Sci..

[15] Washburn R.L., Sandberg D., Gazdik Stofer M.A. (2022). Supplementation of a single species probiotic does not affect diversity and composition of the healthy adult gastrointestinal microbiome. Hum. Nutr. Metab..

[16] Volokh O., Klimenko N., Berezhnaya Y., Tyakht A., Nesterova P., Popenko A., Alexeev D. (2019). Human gut microbiome response induced by fermented dairy product intake in healthy volunteers. Nutrients.

[17] Plaza-Díaz J., Fernández-Caballero J.Á., Chueca N., García F., Gómez-Llorente C., Sáez-Lara M.J., Fontana L., Gil Á. (2015). Pyrosequencing analysis reveals changes in intestinal microbiota of healthy adults who received a daily dose of immunomodulatory probiotic strains. Nutrients..

[18] Kim S.-W., Suda W., Kim S., Oshima K., Fukuda S., Ohno H., Morita H., Hattori M. (2013). Robustness of gut microbiota of healthy adults in response to probiotic intervention revealed by high-throughput pyrosequencing. DNA Res..

[19] Laursen M.F., Laursen R.P., Larnkjær A., Michaelsen K.F., Bahl M.I., Licht T.R. (2017). Administration of two probiotic strains during early childhood does not affect the endogenous gut microbiota composition despite probiotic proliferation. BMC Microbiol..

[20] Wang L., Zhao Z., Zhao L., Zhao Y., Yang G., Wang C., Gao L., Niu C., Li S. (2022). *Lactobacillus plantarum DP189* reduces α-SYN aggravation in MPTP-Induced Parkinson’s disease mice via regulating oxidative damage, inflammation, and gut microbiota disorder. J. Agric. Food Chem..

[21] He Q., Zhang Y., Ma D., Zhang W., Zhang H. (2022). *Lactobacillus casei* Zhang exerts anti-obesity effect to obese glut1 and gut-specific-glut1 knockout mice via gut microbiota modulation mediated different metagenomic pathways. Eur. J. Nutr..

[22] Chen Y.T., Chiou S.Y., Hsu A.H., Lin Y.C., Lin J.S. (2022). *Lactobacillus rhamnosus Strain LRH05* intervention ameliorated body weight gain and adipose inflammation via modulating the gut microbiota in high-fat diet-induced obese mice. Mol. Nutr. Food Res..

[23] Kong C., Akkerman R., Klostermann C.E., Beukema M., Oerlemans M.M.P., Schols H.A., De Vos P. (2021). Distinct fermentation of human milk oligosaccharides 3-FL and LNT2 and GOS/inulin by infant gut microbiota and impact on adhesion of *Lactobacillus plantarum WCFS1* to gut epithelial cells. Food Funct..

[24] Yang F., Zhu W.J., Edirisuriya P., Ai Q., Nie K., Ji X.M., Li Y., Zhou K. (2022). Beneficial effects of a combination of *Clostridium cochlearium* and *Lactobacillus acidophilus* on body weight gain, insulin sensitivity, and gut microbiota in high-fat diet–induced obese mice. Nutrition.

[25] Love M.I., Huber W., Anders S. (2014). Moderated estimation of fold change and dispersion for RNA-seq data with DESeq2. Genome Biol..

[26] Shao J., Li Z., Gao Y., Zhao K., Lin M., Li Y., Wang S., Liu Y., Chen L. (2021). Construction of a “Bacteria-Metabolites” Co-Expression network to clarify the anti-ulcerative colitis effect of flavonoids of sophora flavescens aiton by regulating the “Host-Microbe” interaction. Front. Pharmacol..

[27] Kim Y.S., Milner J.A. (2007). Dietary modulation of colon cancer risk. J. Nutr..

[28] Ulker I., Yildiran H. (2019). The effects of bariatric surgery on gut microbiota in patients with obesity: A review of the literature. Biosci. Microbiota Food Health.

[29] Lindenberg F., Krych L., Fielden J., Kot W., Frøkiær H., van Galen G., Nielsen D.S., Hansen A.K. (2019). Expression of immune regulatory genes correlate with the abundance of specific *Clostridiales* and *Verrucomicrobia* species in the equine ileum and cecum. Sci. Rep..

[30] Shi Y., Zou Y., Xiong Y., Zhang S., Song M., An X., Liu C., Zhang W., Chen S. (2021). Host Gasdermin D restrains systemic endotoxemia by capturing *Proteobacteria* in the colon of high-fat diet-feeding mice. Gut Microbes.

[31] Di Gioia D., Bozzi Cionci N., Baffoni L., Amoruso A., Pane M., Mogna L., Gaggìa F., Lucenti M.A., Bersano E., Cantello R. (2020). A prospective longitudinal study on the microbiota composition in amyotrophic lateral sclerosis. BMC Med..

[32] Hao L., Michaelsen T.Y., Singleton C.M., Dottorini G., Kirkegaard R.H., Albertsen M., Nielsen P.H., Dueholm M.S. (2020). Novel syntrophic bacteria in full-scale anaerobic digesters revealed by genome-centric metatranscriptomics. ISME J..

[33] Huang Y., Wang Z., Ma H., Ji S., Chen Z., Cui Z., Chen J., Tang S. (2021). Dysbiosis and implication of the gut microbiota in diabetic retinopathy. Front. Cell. Infect. Microbiol..

[34] Shin N.R., Whon T.W., Bae J.W. (2015). Proteobacteria: Microbial signature of dysbiosis in gut microbiota. Trends Biotechnol..

[35] Blaser M.J. (2016). Antibiotic use and its consequences for the normal microbiome. Science.

[36] Popenko A.S. (2015). Bioinformatic Study of the Taxonomic Composition of the Human Intestinal Microbiota. Ph.D. Thesis.

[37] Bai Y.F., Wang S.W., Wang X.X., Weng Y.Y., Fan X.Y., Sheng H., Zhu X.T., Lou L.J., Zhang F. (2019). The flavonoid-rich Quzhou Fructus Aurantii extract modulates gut microbiota and prevents obesity in high-fat diet-fed mice. Nutr. Diabetes.

[38] Wang J., Wang Y., Wang S., Cai J., Shi J., Sui X., Cao Y., Huang W., Chen X., Cai Z. (2015). Bone marrow-derived mesenchymal stem cell-secreted IL-8 promotes the angiogenesis and growth of colorectal cancer. Oncotarget.

[39] Erdman S.E., Poutahidis T., Tomczak M., Rogers A.B., Cormier K., Plank B., Horwitz B.H., Fox J.G. (2003). CD4^+^ CD25^+^ regulatory T lymphocytes inhibit microbially induced colon cancer in Rag2-deficient mice. Am. J. Pathol..

[40] Wang J., Lu H., Yu L., Cheng W., Yan W., Jing X. (2021). Aggravation of airway inflammation in RSV-infected asthmatic mice following infection-induced alteration of gut microbiota. Ann. Palliat. Med..

[41] Zhang W., Zhu Y.H., Yang G.Y., Liu X., Xia B., Hu X., Su J.H., Wang J.F. (2018). *Lactobacillus rhamnosus* GG Affects Microbiota and Suppresses Autophagy in the Intestines of Pigs Challenged with Salmonella Infantis. Front. Microbiol..

[42] Plovier H., Everard A., Druart C., Depommier C., Van Hul M., Geurts L., Chilloux J., Ottman N., Duparc T., Lichtenstein L. (2017). A purified membrane protein from *Akkermansia muciniphila* or the pasteurized bacterium improves metabolism in obese and diabetic mice. Nat. Med..

[43] Depommier C., Everard A., Druart C., Plovier H., Van Hul M., Vieira-Silva S., Falony G., Raes J., Maiter D., Delzenne N.M. (2019). Supplementation with *Akkermansia muciniphila* in overweight and obese human volunteers: A proof-of-concept exploratory study. Nat. Med..

[44] Zagato E., Pozzi C., Bertocchi A., Schioppa T., Saccheri F., Guglietta S., Fosso B., Melocchi L., Nizzoli G., Troisi J. (2020). Endogenous murine microbiota member *Faecalibaculum rodentium* and its human homologue protect from intestinal tumour growth. Nat. Microbiol..

[45] Loomba R., Ling L., Dinh D.M., DePaoli A.M., Lieu H.D., Harrison S.A., Sanyal A.J. (2021). The Commensal Microbe *Veillonella* as a Marker for Response to an FGF19 Analog in NASH. Hepatology.

[46] Wang Y., Gao X., Zhang X., Xiao Y., Huang J., Yu D., Li X., Hu H., Ge T., Li D. (2019). Gut Microbiota Dysbiosis Is Associated with Altered Bile Acid Metabolism in Infantile Cholestasis. mSystems.

[47] Weber C. (2015). IBD: *Lactococcus lactis* alleviates oxidative stress and colitis in mice. Nat. Rev. Gastroenterol. Hepatol..

[48] Ai D., Pan H., Li X., Gao Y., Liu G., Xia L.C. (2019). Identifying gut microbiota associated with colorectal cancer using a zero-inflated lognormal model. Front. Microbiol..

[49] Hughes K.R., Harnisch L.C., Alcon-Giner C., Mitra S., Wright C.J., Ketskemety J., Van Sinderen D., Watson A.J.M., Hall L.J. (2017). Bifidobacterium breve reduces apoptotic epithelial cell shedding in an exopolysaccharide and MyD88-dependent manner. Open Biol..

[50] Qi M., Cao Z., Shang P., Zhang H., Hussain R., Mehmood K., Chang Z., Wu Q., Dong H. (2021). Comparative analysis of fecal microbiota composition diversity in Tibetan piglets suffering from diarrheagenic *Escherichia coli* (DEC). Microb. Pathog..

[51] Wei X., Tao J., Xiao S., Jiang S., Shang E., Zhu Z., Qian D., Duan J. (2018). Xiexin Tang improves the symptom of type 2 diabetic rats by modulation of the gut microbiota. Sci. Rep..

[52] Zhang X., Li C., Cao W., Zhang Z. (2021). Alterations of Gastric Microbiota in Gastric Cancer and Precancerous Stages. Front. Cell. Infect. Microbiol..

[53] Pfeiffer N., Desmarchelier C., Blaut M., Daniel H., Haller D., Clavel T. (2012). *Acetatifactor muris* gen. nov., sp. nov., a novel bacterium isolated from the intestine of an obese mouse. Arch. Microbiol..

[54] Lawson P.A., Song Y., Liu C., Molitoris D.R., Vaisanen M.L., Collins M.D., Finegold S.M. (2004). *Anaerotruncus colihominis* gen. nov., sp. nov., from human faeces. Int. J. Syst. Evol. Microbiol..

[55] Olaisen M., Flatberg A., Granlund A.V.B., Røyset E.S., Martinsen T.C., Sandvik A.K., Fossmark R. (2021). Bacterial Mucosa-associated Microbiome in Inflamed and Proximal Noninflamed Ileum of Patients with Crohn’s Disease. Inflamm. Bowel Dis..

[56] Göker M., Gronow S., Zeytun A., Nolan M., Lucas S., Lapidus A., Hammon N., Deshpande S., Cheng J.F., Pitluck S. (2011). Complete genome sequence of *Odoribacter splanchnicus* type strain (1651/6). Stand. Genom. Sci..

[57] Morgan X.C., Tickle T.L., Sokol H., Gevers D., Devaney K.L., Ward D.V., Reyes J.A., Shah S.A., LeLeiko N., Snapper S.B. (2012). Dysfunction of the intestinal microbiome in inflammatory bowel disease and treatment. Genome Biol..

[58] Kraler M., Ghanbari M., Domig K.J., Schedle K., Kneifel W. (2016). The intestinal microbiota of piglets fed with wheat bran variants as characterised by *16S rRNA* next-generation amplicon sequencing. Arch. Anim. Nutr..

[59] Okeke F., Roland B.C., Mullin G.E. (2014). The role of the gut microbiome in the pathogenesis and treatment of obesity. Glob. Adv. Health Med..

[60] Parker B.J., Wearsch P.A., Veloo A.C.M., Rodriguez-Palacios A. (2020). The Genus *Alistipes*: Gut Bacteria with Emerging Implications to Inflammation, Cancer, and Mental Health. Front. Immunol..

[61] Schaedler R.W., Dubos R., Costello R. (1965). The development of the bacterial flora in the gastrointestinal tract of mice. J. Exp. Med..

[62] Westerman L.J., De Boer R.F., Roelfsema J.H., Friesema I.H.M., Kortbeek L.M., Wagenaar J.A., Bonten M.J.M., Kusters J.G. (2013). *Brachyspira* species and gastroenteritis in humans. J. Clin. Microbiol..

[63] Alessandri G., Ossiprandi M.C., MacSharry J., van Sinderen D., Ventura M. (2019). Bifidobacterial Dialogue with Its Human Host and Consequent Modulation of the Immune System. Front. Immunol..

[64] Fritz B., Bier-Kirkegaard J., Nielsen C.H., Kirketerp-Møller K., Malone M., Bjarnsholt T. (2021). Transcriptomic Fingerprint of Bacterial Infection in Lower Extremity Ulcers. medRxiv.

[65] Sims G.E., Kim S.H. (2011). Whole-genome phylogeny of *Escherichia coli*/*Shigella* group by feature frequency profiles (FFPs). Proc. Natl. Acad. Sci. USA.

