# Supplementary material for: Dynamics of Changes in the Gut Microbiota of Healthy Mice Fed with Lactic Acid Bacteria and Bifidobacteria
Source: Microorganisms. 2022 May 12;10(5):1020. doi: 10.3390/microorganisms10051020 (PMC9144108; doi:10.3390/microorganisms10051020)
Supplement: Supplementary file 1 [file microorganisms-10-01020-s001.zip › Supplementary File S2_Diet composition.pdf]

# Diet composition

**Crude Nutrients [%]**
Crude protein (N x 6.25) 23.0
Crude fat 6.1
Crude fibre 3.3
NDF 11.3
ADF 5.2
Crude ash 6.5
Starch 34.1
Sugar 5.1
N free extracts 49.8

**Minerals [%]**
Calcium 1.00
Phosphorus 0.70
Ca/P 1.43:1
Sodium 0.23
Magnesium 0.22
Potassium 1.03

**Fatty acids [%]**
C 12:0 -
C 14:0 0.01
C 16:0 0.68
C 18:0 0.22
C 20:0 0.03
C 16:1 0.04
C 18:1 1.47
C 18:2 3.23
C 18:3 0.37

**Amino acids [%]**
Lysine 1.57
Methionine 0.54
Cystine 0.40
Met+Cys. 0.94
Threonine 0.89
Tryptophan 0.29

Arginine 1.50
Histidine 0.61
Valine 1.12
Isoleucine 1.02
Leucine 1.79
Phenylalanine 1.12
Phe+Tyr 1.91
Glycine 1.05
Glutamic acid 4.83
Aspartic acid 2.42
Proline 1.50
Serine 1.26
Alanine 1.08

**Vitamins per kg**
Vitamin A 25,000 IU
Vitamin D 3 1,500 IU
Vitamin E 135 mg
Vitamin K (as MNB) 20 mg
Thiamine (B;) 86 mg
Riboflavin (B2) 33 mg
Pyridoxine (Bs) 32 mg
Cobalamin (B;2) 150 μg
Nicotinic acid 140 mg
Pantothenic acid 58 mg
Folic acid 10 mg
Biotin 690 μg
Choline 1,400 mg

**Trace elements per kg**
Iron 180 mg
Manganese 58 mg
Zinc 86 mg
Copper 15 mg
lodine 2.1 mg
Selenium 0.3 mg
